# Preparation and quality evaluation of potato steamed bread with wheat gluten

**DOI:** 10.1002/fsn3.1600

**Published:** 2020-06-23

**Authors:** Beibei Zhao, Jiawen Deng, Mingyue Li, Hua Li, Yan Zhang, Haodi Gong, Zhicheng Chen

**Affiliations:** ^1^ College of Food Science and Engineering Henan University of Technology Zhengzhou China

**Keywords:** potato flour, sensory evaluation, steamed bread, texture

## Abstract

The present study aimed to study the preparation and quality evaluation of potato steamed bread by using potato flour, wheat flour, and gluten at the presence of yeast and inorganic additives. As the rheological properties of the potato–wheat formulated flour negatively related to the potato flour, the potato–wheat formulated flour with 35% potato flour was set as the basic flour (100%). The effects of wheat gluten on the rheological properties of the dough were also evaluated, and gluten addition amount was set at 6.5%. The effects of yeast, sodium bicarbonate, citric acid, and monocalcium phosphate addition on steamed bread properties have been studied and optimized by orthogonal test. The obtained potato steamed bread formula was 100% basic flour (potato/wheat mass ratio of 35:65), 6.5% wheat gluten, 1.1% yeast, 1.4% NaHCO_3_, 0.75% citric acid, and 0.50% Ca(H_2_PO_4_)_2_. The prepared potato steamed bread has good sensory and texture properties, with natural potato flavor.

## Graphical abstract

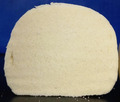

## 1 INTRODUCTION

Chinese steamed bread is one of the important staple foods in China. Traditional steamed bread formula was wheat flour, water, and yeast or sourdough. Nowadays, wholegrain and multigrain foods have become a new trend to improve the nutritional value of food (Angioloni & Collar, 2011; Osuna, Romero, Romero, Judis, & Bertola, 2018).

Potato (*Solanum tuberosum*) is the fourth major food crop in the world after wheat, rice, and corn. With cultivation area 19.3 million ha, worldwide production of potato was estimated to be 388.2 million tons in 2017 (FAOSTAT, 2019). Compared with wheat flour, potato flour contains relatively higher dietary fiber, mineral elements, and chemical compounds with antioxidant activity, such as carotene, folic acid, chlorogenic acid, and anthocyanin (Liu et al., 2017). The estimated glycemic index of potato foods was relatively lower than that of wheat foods (Zhu, 2019). The effect of potato flour on dough rheological properties has been studied, and the results showed that replacing wheat flour with potato flour reduced dough strength significantly (Liu, Mu, Sun, Zhang, & Chen, 2016; Pu et al., 2017). Substitution of potato flour for wheat flour in many cereal products such as noodles, bread, steamed bread, and cookies has been investigated (Dankwa, Liu, & Pu, 2017; Zhang, Xu, Wu, Hu, & Dai, 2017; Zhao, Mu, & Sun, 2019). It has been reported that potato flour substitution at 6% in bread making was acceptable (Zeng et al., 2019). Potato flour replaces of wheat flour in steamed bread making is still a challenge. Liu reported that replacement of wheat flour up to 35% was acceptable for steamed bread, but hydroxypropyl methylcellulose (HPMC) and xanthan gum had been used to improve the processing properties (Liu et al., 2018). Another formulation contained HPMC and egg white protein has also been reported (Liu, Mu, et al., 2019). However, there was no study about the potato steamed bread with wheat gluten.

In this study, the preparation and quality evaluation of potato steamed bread with wheat gluten have been studied. The influence of potato flour ratio on the rheological properties of the dough has been studied. With a fixed potato/wheat mass ratio, the effect of wheat gluten on rheological properties of the dough was evaluated. The influences of yeast, sodium bicarbonate, citric acid, and monocalcium phosphate addition on steamed bread properties have been studied and optimized by orthogonal test. At last, a new potato steamed bread formula by using potato flour, wheat flour, and gluten at the presence of yeast, NaHCO_3_, citric acid, and Ca(H_2_PO_4_)_2_ has been developed.

## 2 MATERIALS AND METHODS

### 2.1 Materials

Commercial wheat flour (moisture 14.4%, protein 11.6%, and ash 0.42%) and potato flour (moisture 7.3%, protein 8.3%, and ash 2.4%) were purchased from Jinyuan Co., Ltd and Fuguang Co., Ltd, respectively. Wheat gluten was supplied by Zhengte Co., Ltd. Yeast, NaHCO_3_, citric acid, and Ca(H_2_PO_4_)_2_ were of food grade, and other chemical reagents were of analytical grade.

### 2.2 Flour blending

The potato/wheat mass ratio was determined as 0:100, 5:95, 10:90, 15:85, 20:80, 25:75, 30:70, 35:65, and 40:60, referred as S0, S5, S10, S15, S20, S25, S30, S35, and S40, respectively. Potato flour and wheat flour were, respectively, weighed according to the proportion and then thoroughly incorporated in the mixer and sealed preserved at 4°C.

The formulated flour of S35 with a potato mass ratio of 35% was selected to be the basic flour. Gluten was added to the basic flour at levels of 6.0%, 6.5%, and 7.0%, referred as S35+W6.0, S35+W6.5, and S35+W7.0, respectively. The basic flour and gluten were, respectively, weighted according to the proportion and then thoroughly incorporated in the mixer and sealed preserved at 4°C.

### 2.3 Chemical analysis

Before steamed bread preparation, chemical composition and rheological properties of the potato–wheat formulated flour samples (S0, S5, S10, S15, S20, S25, S30, S35, and S40) and basic flour–gluten formulated flour samples (S35+W6.0, S35+W6.5, and S35+W7.0) were tested. Moisture, ash, protein, and gluten index were determined according to the AACC methods 44‐15A, 08‐21.01, 46‐12.01, and 38‐12.02, respectively (AACC, 2000). Farinograph test was performed according to the ICC method No.115/1 by Farinograph‐AT (Brabender) (ICC, 2010). Extensograph test was carried out according to the ICC method No. 114/1 by Extensograph‐E (Brabender) (ICC, 2010).

### 2.4 Steamed bread preparation

#### 2.4.1 Preparation method

The production of steamed bread followed a previous method with some modification (Zhu, 2014). The formulated flour of S35 with potato mass ratio of 35% was selected to be the basic flour, and the gluten addition amount was set at 6.5%. First, 100 g of the based flour S35 (65 g of the wheat flour and 35 g of the potato flour), 6.5 g of gluten, 1.1 g of NaHCO_3_, 0.6 g of citric acid, and 0.3 g of Ca(H_2_PO_4_)_2_ were mixed homogeneously. Second, 1.0 g of dried yeast was dispersed in 50 g distilled water (40°C) for 10 min before mixing with the flour. Then, the dough was prepared by mixing the ingredients in a Swanson mixer (National Co., Ltd) at a speed of 105 rpm for 5 min. The dough was sheeted for ten times on a noodle machine, hand‐kneaded for 1 min, and shaped. The proofed dough was fermented at 37°C and 55% relative humidity for 40 min in a controlled fermentation cabinet (MXF‐A, Mingsheng), then steamed in a steaming chamber for 20 min, and cooled at room temperature for 0.5 hr before evaluation.

#### 2.4.2 Single factor experiment

With fixed basic flour amount of 100% and gluten addition amount of 6.5%, the effects of inorganic additives on steamed bread properties were studied. As Table 1 shown, four factors were selected: yeast, NaHCO_3_, citric acid, and Ca(H_2_PO_4_)_2_ addition.

**TABLE 1:** Inorganic addition amounts of potato steamed bread

Types of steamed bread	Addition amount (%)			
	Yeast	NaHCO_3_	Citric acid	Ca(H_2_PO_4_)_2_
SB‐YEA‐1	0.8	1.1	0.6	0.3
SB‐YEA‐2	0.9	1.1	0.6	0.3
SB‐YEA‐3	1.0	1.1	0.6	0.3
SB‐YEA‐4	1.1	1.1	0.6	0.3
SB‐YEA‐5	1.2	1.1	0.6	0.3
SB‐SB‐1	1.0	0.9	0.6	0.3
SB‐SB‐2	1.0	1.1	0.6	0.3
SB‐SB‐3	1.0	1.3	0.6	0.3
SB‐SB‐4	1.0	1.5	0.6	0.3
SB‐SB‐5	1.0	1.7	0.6	0.3
SB‐CA‐1	1.0	1.1	0.5	0.3
SB‐CA‐2	1.0	1.1	0.6	0.3
SB‐CA‐3	1.0	1.1	0.7	0.3
SB‐CA‐4	1.0	1.1	0.8	0.3
SB‐CA‐5	1.0	1.1	0.9	0.3
SB‐MP‐1	1.0	1.1	0.6	0.1
SB‐MP‐2	1.0	1.1	0.6	0.2
SB‐MP‐3	1.0	1.1	0.6	0.3
SB‐MP‐4	1.0	1.1	0.6	0.4
SB‐MP‐5	1.0	1.1	0.6	0.5

Note

SB‐YEA‐1 to SB‐YEA‐5: single factor experiments of yeast amount, SB‐SB‐1 to SB‐SB‐5: single factor experiments of NaHCO_3_ amount, SB‐CA‐1 to SB‐CA‐5: single factor experiments of citric acid amount, SB‐MP‐1 to SB‐MP‐5: single factor experiments of Ca(H_2_PO_4_)_2_.

#### 2.4.3 Orthogonal test

As Table 2 shown, the effects of inorganic additives on steamed bread properties were then optimized by four‐factor and three‐level orthogonal test. Specific volume determination and sensory evaluation were conducted.

**TABLE 2:** Orthogonal test results and range analysis

No.	A Yeast (%)	B NaHCO_3_ (%)	C Citric acid (%)	D Ca(H_2_PO_4_)_2_ (%)	Specific volume (mL/g)	Sensory score
						(100)
1	0.9	1.3	0.65	0.40	1.66	80.2
2	0.9	1.4	0.70	0.45	1.87	77.3
3	0.9	1.5	0.75	0.50	2.19	86.8
4	1.0	1.3	0.70	0.50	1.89	85.4
5	1.0	1.4	0.75	0.40	2.2	89.3
6	1.0	1.5	0.65	0.45	2.27	71.7
7	1.1	1.3	0.75	0.45	1.75	86.5
8	1.1	1.4	0.65	0.50	1.93	95.0
9	1.1	1.5	0.70	0.4	2.14	70.0
Specific volume						
Average 1	1.91	1.77	1.95	1.98	Sequence: B > A>C > D	Optimal combination: A_2_B_3_C_3_D_3_
Average 2	2.12	2.00	1.97	1.96		
Average 3	1.94	2.20	2.05	2.00		
Range	0.21	0.43	0.10	0.04		
Sensory score						
Average 1	81.4	84.0	82.3	24.8	Sequence: B > D>C > A	Optimal combination: A_3_B_2_C_3_D_3_
Average 2	82.2	87.2	77.6	78.5		
Average 3	83.8	76.2	87.6	89.1		
Range	2.4	11.0	10.0	10.6		

Note

Average 1, average 2, and average 3 are the mean values calculated by L9(34) orthogonal design method. Range value = Average_max_ − Average_min_.

### 2.5 Steamed bread evaluation

#### 2.5.1 Specific volume determination

The cooled steamed bread was weighed, and its volume was measured by the millet replacement method. The specific volume of the steamed bread was the result of volume divided by weight.

#### 2.5.2 Sensory evaluation

Sensory evaluation of the steamed bread was carried out by ten trained panelists from Henan University of Technology according to the Chinese standard method SB/T 10139‐1993 with some modification (Liu, Solah, et al., 2019). Sensory items are shown in Table 3.

**TABLE 3:** Sensory evaluation for potato steamed bread

Quality parameters	Score	Evaluation rules
Specific volume	20	2.3 ml/g (20), and one point will be deducted for every 0.1 ml/g less specific volume
Appearance		
Shape	10	Good volume and symmetry, good upright (8–10) Basic symmetry and upright (4–7) No symmetry, low height, or bad shape (1–3)
Smoothness	5	Very smooth, bright, and no specks (4–5) Rough surface, shrinking skin, specks, or bubbles in skin (1–3)
Color	10	White/creamy white (8–10), little yellow (4–7), gray or dark (1–3)
Interior texture		
Visual internal texture	15	Good crumb structure dense, homogenous, and spongy (no big holes) (11–15), acceptable crumb, homogenous with few big holes (6–10), poor, uneven large holes, not homogenous (1–5)
Elasticity/firmness by touch and bite	10	Good bounce back when pressed with finger and bite a little hard, a little stress or toughness (8–10), bounce back slowly and bite with a little stress (4–7), poor no bounce and crumbly (1–3)
Stickiness (mouthfeel)	10	Not sticky (8–10), a little sticky (4–7), poor very sticky (1–3)
Texture in mouth	10	Not rough/fine (8–10), little rough (4–7), poor very rough (1–3)
Aroma		
Aroma/flavor	10	Good fresh wheat and potato smell (8–10), not fresh wheat and potato smell (4–7), abnormal or musty smell (1–3)

#### 2.5.3 Texture analysis of steamed bread

The texture analysis was conducted on the steamed bread after steaming for 20 min and stored at room temperature for 1 hr. The texture properties of the steamed bread were analyzed as described by using a TA‐XT2i Texture Analyzer equipped with a probe of P/36R (Stable Microsystems). Texture profile analysis (TPA) was set as: pre‐ and post‐test speed of 2.0 mm/s, test speed of 1.0 mm/s, trigger force of 5 g, compression distance of 25 mm, and pause between two compression of 1 s. Hardness, stickiness, springness, cohesiveness, chewiness, and resilience were measured.

### 2.6 Statistical analysis

All experiments were repeated in triplication, and results were expressed as mean ± *SD*. The ANOVA procedure and Duncan's multiple range method were used to evaluate the significant differences between treatments (*p* < .05).

## 3 RESULTS AND DISCUSSION

### 3.1 Mixing behavior of the formulated flour

**FIGURE 1:**
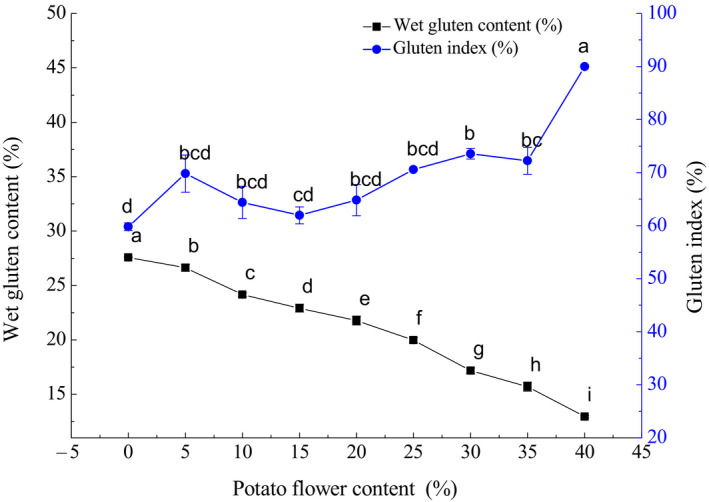
Wet gluten content and gluten index of the formulated flour with different potato flower content

Chemical composition of the formulated flour was characterized in our study (Table S1). As Figure 1 shown, the wet gluten content in the formulated flour declined as the mass ratio of potato flour increased, due to no gluten existed in potato flour (Xu, Hu, Liu, Dai, & Zhang, 2017). But the gluten index gradually increased at the ratio increased, indicating improvement of the gluten flexibility. Table 4 shows the farinograph parameters of the formulated flour. The water absorption rate of the formulated flour increased with the potato ratio, because of the stronger water absorption ability of potato flour. It has been reported that the water absorption rate of potato flour was 148%, much higher than that of the wheat flour (62.8%) (Zeng et al., 2019).

**TABLE 4:** Farinograph parameters of the formulated flour

Sample	Wet gluten content (%)	Water absorption (%)	Formation time (min)	Stability time (min)	Weakness (FU)	Evaluation score
S0	28.0^a^	59.3^i^	2.83^g^	6.63^a^	68^f^	79^a^
S5	26.6^b^	65.7^h^	2.43^h^	4.31^b^	198^e^	58^b^
S10	24.2^c^	74.7^g^	2.43^h^	2.46^e^	272^d^	49^c^
S15	22.9^d^	82.0^f^	3.70^e^	2.45^e^	293^b^	45^d^
S20	21.8^e^	88.8^e^	3.33^f^	1.93^h^	317^a^	41^e^
S25	20.0^f^	94.4^d^	3.76^d^	2.30^f^	295^b^	32^f^
S30	17.2^g^	96.2^c^	3.82^c^	2.21^g^	287^c^	30^g^
S35	15.7^h^	97.9^b^	4.26^a^	3.15^d^	294^b^	27^h^
S40	12.9^i^	100.4^a^	4.11^b^	3.33^c^	295^b^	20^i^
S35+W5.0	26.5^e^	101.2^e^	4.98^e^	3.42^e^	287^a^	52^e^
S35+W5.5	27.7^d^	101.7^d^	5.04^d^	3.47^d^	282^b^	58^d^
S35+W6.0	28.2^c^	102.3^c^	5.09^c^	3.51^c^	278^c^	63^c^
S35+W6.5	29.0^b^	103.0^b^	5.14^b^	3.55^b^	270^d^	67^a^
S35+W7.0	29.7^a^	105.5^a^	5.20^a^	3.70^a^	282^b^	65^b^

Note

S0, S5, S10, S15, S20, S25, S30, S35, and S40 refer to formulated potato/wheat flour blends samples with potato/wheat mass ratio of 0:100, 5:95, 10:90, 15:85, 20:80, 25:75, 30:70, 35:65, and 40:60, respectively. S35+W5.0, S35+W5.5, S35+W6.0, S35+W6.5, and S35+W7.0 refer to samples of the base flour (S35) with gluten addition levels of 5.0%, 5.5%, 6.0%, 6.5%, and 7.0%, respectively. Results are presented as mean value ± *SD*. Different letters within a column mean significant difference (*p* < .05).

Compared with the control (2.83 min), the formation time of the formulated flour with 5% (2.43 min) or 10% potato flour (2.43 min) significantly decreased. But further increase of the potato ratio raised the formation time of the formulated flour. For instance, the formation time of the formulated flour with 35% potato flour was 4.26 min. The literature revealed that blending potato flour with wheat flour needed more time for the dough to reach the maximum consistency (Laukova, Minarovicova, Karovicova, & Kohajdova, 2019).

The wheat protein was high in glutenin and gliadin, which were responsible for the dough strength, while the potato protein was mainly composed of globulin and alkali‐soluble protein (Muneer, Johansson, Hedenqvist, Plivelic, & Kuktaite, 2019). The gluten content of the formulated flour decreased with the increase of the potato mass ratio, resulting in decline of the dough strength. As Table 4 shown, the dough stability times of the formulated flour with different contents of potato flour were significantly lower than that of the control (6.63 min). However, as the ratio increased, the stability time first decreased and then increased. Pu reported it has been related to the change of water absorption and the uniformity of the formulated flour (Pu et al., 2017). Meanwhile, as the ratio increased, the weakness exhibited an increasing tendency, and the evaluation score declined significantly (Table 4).

The farinograph properties of the formulated flour are shown in Table 5. As the mass ratio of potato flour increased, the extensible area reduced from 78.0 to 9.0 cm^2^, and the extensibility of the flour decreased from 164.0 to 113.1 mm. Also, the extensible resistance and the max‐extensible resistance reduced significantly. But the extensible rate and the max‐extensible rate first decreased and then increased as the ratio increased. The results indicated decline of the dough strength and degradation of the processing properties of the dough.

**TABLE 5:** Extensograph parameters of the formulated flour

Sample	Extensible area (cm^2^)	Extensibility (mm)	Extensible resistance (BU)	Extensible rate	Max‐extensible resistance (BU)	Max‐extensible rate
S0	78.0^a^	164.0^a^	310.0^a^	2.00^a^	379.0^a^	2.60^a^
S5	62.0^b^	146.0^b^	262.0^b^	1.80^b^	248.0^b^	2.50^b^
S10	35.6^c^	147.0^b^	157.7^c^	1.07^f^	199.0^c^	1.10^h^
S15	30.0^d^	142.0^c^	140.4^d^	0.99^g^	140.0^d^	1.50^d^
S20	16.0^f^	135.5^d^	134.0^e^	0.98^g^	106.5^g^	0.99^i^
S25	19.0^e^	130.0^e^	126.0^f^	0.97^g^	132.0^e^	1.13^f^
S30	13.0^g^	129.7^e^	121.0^g^	1.16^e^	108.5^f^	1.24^f^
S35	11.0^h^	120.8^f^	111.9^h^	1.34^d^	102.0^h^	1.32^e^
S40	9.0^i^	113.1^g^	106.3^i^	1.56^c^	99.6^i^	1.53^c^
S35+W5.0	19.7^e^	131.5^e^	123.2^e^	1.03^a^	129.7^e^	1.16^b^
S35+W5.5	22.1^d^	135.7^d^	126.9^d^	1.01^b^	134.6^d^	1.14^c^
S35+W6.0	24.9^c^	140.3^c^	130.7^c^	0.98^c^	140.8^c^	1.10^e^
S35+W6.5	27.4^b^	144.4^b^	136.3^b^	0.94^d^	147.3^b^	1.12^d^
S35+W7.0	30.7^a^	149.8^a^	145.5^a^	1.00^b^	162.0^a^	1.18^a^

Note

S0, S5, S10, S15, S20, S25, S30, S35, and S40 refer to formulated potato/wheat flour blends samples with potato/wheat mass ratio of 0:100, 5:95, 10:90, 15:85, 20:80, 25:75, 30:70, 35:65, and 40:60, respectively. S35+W5.0, S35+W5.5, S35+W6.0, S35+W6.5, and S35+W7.0 refer to samples of the base flour (S35) with gluten addition levels of 5.0%, 5.5%, 6.0%, 6.5%, and 7.0%, respectively. Results are presented as mean value ± *SD*. Different letters within a column mean significant difference (*p* < .05).

### 3.2 Effect of gluten on formulated flour

Addition of gluten in low protein flour has been proved to be able to improve viscoelasticity and extensibility of the dough (Ma & Baik, 2017). In this study, the formulated flour with 35% potato flour and 65% wheat flour was selected to be the basic flour. Effects of gluten (5.0%–7.0%) on the farinograph and extensograph properties of the dough have been studied.

As Table 4 shown, as the gluten addition amount increased, farinograph properties of the dough showed an increasing tendency. According to the requirements of SB/T10137‐93 for making steamed bread, wet gluten content of the flour should be 28%–30%, and stability time of the dough should be higher than 3 min. With gluten addition of 6.0%–7.0%, the wet gluten content of the flour reached 28.2%–29.7%, and the stability time reached 3.51–3.70 min. Therefore, the gluten addition amount of 6.0%–7.0% was feasible.

Compared with formulated flour S35, the addition of gluten also significantly improved the extensograph properties of the flour (Table 5). With gluten addition of 6.5%, the extensible area reached 27.4 cm^2^, and the extensibility reached 144.4 mm. Also, the extensible and max‐extensible resistance reached 136.3 BU and 147.3 BU, respectively, significantly higher than those of the formulated flour S35.

### 3.3 Steamed bread sensory properties

With potato–wheat formulated flour S35 as the basic flour (100%), and the gluten addition amount was set at 6.5%. The effects of yeast, sodium bicarbonate, citric acid, and monocalcium phosphate additions on steamed bread properties have been studied.

#### 3.3.1 Effect of yeast on steamed bread

As shown in Table 6, with the increase of the yeast amount, the specific volume of the steamed bread gradually increased, while the scores of appearance, interior texture, and aroma all first increased and then decreased. The maximum sensory score (88.5) was obtained at 1.0% addition. During the dough fermentation, the CO_2_ produced by yeast fermentation made the dough to be looser, resulting in increase of the specific volume. However, excessive CO_2_ would lead to formation of so many irregular holes, broken of the wheat gluten network, even collapse or deformation of the steamed bread (Gao, Tay, Koh, & Zhou, 2017). Therefore, the yeast addition of 1.0%–1.1% was feasible.

**TABLE 6:** Effects of inorganic additions on steamed bread sensory parameters

Sample	Specific volume	Shape	Smoothness	Color	Visual internal structure	Elasticity/firmness	Stickiness	Texture in mouth	Aroma	Total score
SB‐YEA‐1	16.5 ± 0.2	9.0 ± 0.2	4.5 ± 0.0	7.8 ± 0.2	11.7 ± 0.2	6.8 ± 0.0	7.5 ± 0.1	8.3 ± 0.1	7.2 ± 0.1	79.5 ± 0.5
SB‐YEA‐2	18.3 ± 0.3	9.1 ± 0.2	4.6 ± 0.1	8.3 ± 0.1	13.2 ± 0.3	7.4 ± 0.1	8.2 ± 0.2	8.6 ± 0.1	7.4 ± 0.2	85.0 ± 0.6
SB‐YEA‐3	18.5 ± 0.1	9.2 ± 0.1	4.6 ± 0.0	9.0 ± 0.2	13.5 ± 0.1	7.8 ± 0.3	8.3 ± 0.1	8.9 ± 0.2	8.8 ± 0.3	88.5 ± 0.7
SB‐YEA‐4	18.6 ± 0.2	9.0 ± 0.3	4.5 ± 0.1	9.0 ± 0.3	14.2 ± 0.4	7.8 ± 0.2	8.5 ± 0.3	9.0 ± 0.3	7.1 ± 0.2	87.7 ± 0.4
SB‐YEA‐5	18.9 ± 0.3	9.0 ± 0.1	4.5 ± 0.0	8.8 ± 0.1	14.1 ± 0.3	7.7 ± 0.1	8.5 ± 0.1	8.8 ± 0.2	6.4 ± 0.1	86.8 ± 0.6
SB‐SB‐1	16.5 ± 0.2	9.0 ± 0.0	4.5 ± 0.2	8.5 ± 0.0	12.7 ± 0.2	7.4 ± 0.1	7.9 ± 0.1	8.6 ± 0.0	8.2 ± 0.2	83.5 ± 0.5
SB‐SB‐2	18.5 ± 0.3	9.2 ± 0.2	4.6 ± 0.0	9.0 ± 0.3	13.5 ± 0.4	7.8 ± 0.2	8.3 ± 0.2	8.9 ± 0.1	8.8 ± 0.1	88.5 ± 0.8
SB‐SB‐3	18.8 ± 0.1	9.3 ± 0.1	4.6 ± 0.1	9.7 ± 0.2	13.8 ± 0.5	8.2 ± 0.1	8.6 ± 0.3	9.3 ± 0.2	9.2 ± 0.2	91.5 ± 1.1
SB‐SB‐4	19.3 ± 0.1	9.5 ± 0.1	4.8 ± 0.1	8.8 ± 0.1	14.3 ± 0.2	8.4 ± 0.2	8.8 ± 0.3	9.2 ± 0.1	8.2 ± 0.2	91.1 ± 1.0
SB‐SB‐5	19.5 ± 0.2	9.6 ± 0.3	4.8 ± 0.0	8.4 ± 0.2	14.3 ± 0.1	8.4 ± 0.2	8.6 ± 0.1	9.1 ± 0.2	7.8 ± 0.1	90.6 ± 0.6
SB‐CA‐1	17.9 ± 0.3	9.2 ± 0.1	4.6 ± 0.1	9.0 ± 0.2	12.7 ± 0.1	7.4 ± 0.1	7.9 ± 0.1	8.6 ± 0.1	7.7 ± 0.1	84.9 ± 0.6
SB‐CA‐2	18.5 ± 0.2	9.2 ± 0.3	4.6 ± 0.2	9.0 ± 0.1	13.5 ± 0.4	7.8 ± 0.2	8.3 ± 0.2	8.9 ± 0.2	8.8 ± 0.2	88.5 ± 0.7
SB‐CA‐3	18.1 ± 0.2	9.4 ± 0.3	4.6 ± 0.1	9.1 ± 0.3	13.9 ± 0.2	8.2 ± 0.1	8.7 ± 0.3	9.2 ± 0.1	9.2 ± 0.3	90.3 ± 0.7
SB‐CA‐4	18.3 ± 0.2	9.5 ± 0.1	4.7 ± 0.0	9.0 ± 0.2	14.2 ± 0.5	8.4 ± 0.3	8.8 ± 0.2	9.3 ± 0.3	6.6 ± 0.1	88.7 ± 0.5
SB‐CA‐5	18.2 ± 0.2	9.5 ± 0.2	4.7 ± 0.2	9.0 ± 0.1	14.2 ± 0.3	8.4 ± 0.2	8.4 ± 0.1	9.2 ± 0.2	5.0 ± 0.1	86.6 ± 0.5
SB‐MP‐1	17.5 ± 0.2	9.1 ± 0.2	4.6 ± 0.1	8.8 ± 0.0	12.7 ± 0.1	6.9 ± 0.1	7.5 ± 0.1	7.7 ± 0.1	8.2 ± 0.1	82.9 ± 0.4
SB‐MP‐2	17.8 ± 0.3	9.2 ± 0.1	4.6 ± 0.1	8.8 ± 0.2	13.3 ± 0.2	7.3 ± 0.1	7.9 ± 0.2	7.8 ± 0.2	8.9 ± 0.2	85.4 ± 0.6
SB‐MP‐3	18.5 ± 0.1	9.2 ± 0.2	4.6 ± 0.2	9.0 ± 0.3	13.5 ± 0.3	7.8 ± 0.2	8.3 ± 0.2	8.9 ± 0.3	8.8 ± 0.1	88.5 ± 0.6
SB‐MP‐4	18.5 ± 0.2	9.1 ± 0.0	4.6 ± 0.0	8.8 ± 0.2	14.2 ± 0.3	7.9 ± 0.3	8.6 ± 0.3	8.8 ± 0.3	9.0 ± 0.2	89.4 ± 0.7
SB‐MP‐5	18.4 ± 0.2	9.4 ± 0.3	4.7 ± 0.1	8.8 ± 0.2	14.1 ± 0.4	7.9 ± 0.3	8.6 ± 0.2	8.8 ± 0.1	8.1 ± 0.0	88.7 ± 0.7

Note

Formulation of each sample refers to Table 1. Results are presented as mean value ± *SD*. Different letters within a column mean significant difference (*p* < .05).

#### 3.3.2 Effect of NaHCO_3_ on steamed bread

The effect of NaHCO_3_ on sensory properties of steamed bread was studied. As Table 6 shown, the specific volume, scores of appearance, and texture of the steamed bread increased with the NaHCO_3_ addition amount. However, when NaHCO_3_ addition amount was higher than 1.3%, the color, texture in mouth, and aroma qualities of the steamed bread became worse. For instance, with NaHCO_3_ addition of 1.5% (Sample SB‐SB‐4), the color of the steamed bread became yellower, unpleasant alkali aroma could be detected, and the aroma of the potato flour and the steamed bread fermentation could not be detected. NaHCO_3_ can react with organic acid produced by lactic acid bacteria during proofing and steaming, at the same time producing more gas (Guo, Yang, & Zhu, 2019). It has been reported that NaHCO_3_ addition on steamed bread could promote protein cross‐linking, therefore improving the strength of the gluten network and enhancing gas retention ability of the dough (Li, Guo, Zhu, & Zhou, 2018). Therefore, the NaHCO_3_ addition of 1.3% was feasible for potato spontaneous steamed bread formula.

#### 3.3.3 Effect of citric acid on steamed bread

In this study, the effect of citric acid addition (0.5%–0.9%) on sensory properties of steamed bread was tested. The results showed that there was no significant difference in specific volume and appearance score between steamed breads with different addition amounts of citric acid. The aroma score firstly increased and then decreased as the citric acid amount increased. With addition of 0.8% or 0.9%, the steamed bread had an unpleasant sour flavor. It has been reported that citric acid in steamed bread could not only enhance the taste property of the steamed bread, but also prolong the retention period of the steamed bread with good antioxidant ability (Blanco, Ronda, Perez, & Pando, 2011; Su et al., 2019). As the highest score was obtained at 0.7% addition (90.3), that amount was thought to be suitable.

#### 3.3.4 Effect of Ca(H_2_PO_4_)_2_ on steamed bread

As Ca(H_2_PO_4_)_2_ addition increased from 0.1% to 0.3%, the specific volume increased gradually. But further increase of the addition did not raise the specific volume value. With Ca(H_2_PO_4_)_2_ addition of 0.5%, both shape and smoothness scores reached the maximum value. Phosphate has three hydrogen bond positions (＝O, －OH, and －OH), producing stronger bonding through the negatively charged oxygen to prevent CO_2_ escape. Phosphate has been reported to be able to enhance both the specific volume and the texture parameters of the bread (Blanco et al., 2011). The highest sensory score of 89.4 was obtained with Ca(H_2_PO_4_)_2_ addition of 0.4%. Therefore, the appropriate Ca(H_2_PO_4_)_2_ addition amount was 0.4%.

### 3.4 Optimization of the steamed bread preparation

Optimization of the steamed bread preparation was obtained by the L9(34) orthogonal design method, as Table 2 shown. Two evaluation indexes were selected, specific volume and sensory score of steamed bread. If specific volume was taken as the evaluation index, the effects sequence was: NaHCO_3_ (B) > yeast (A) > citric acid (C) > Ca(H_2_PO_4_)_2_ (D), and the optimal combination was A_2_B_3_C_3_D_3_. If sensory score was taken as the evaluation index, the effects sequence was: NaHCO_3_ (B) > Ca(H_2_PO_4_)_2_ (D) > citric acid (C) > yeast (A), and optimal combination was A_3_B_2_C_3_D_3_.

### 3.5 Model validation

**FIGURE 2:**
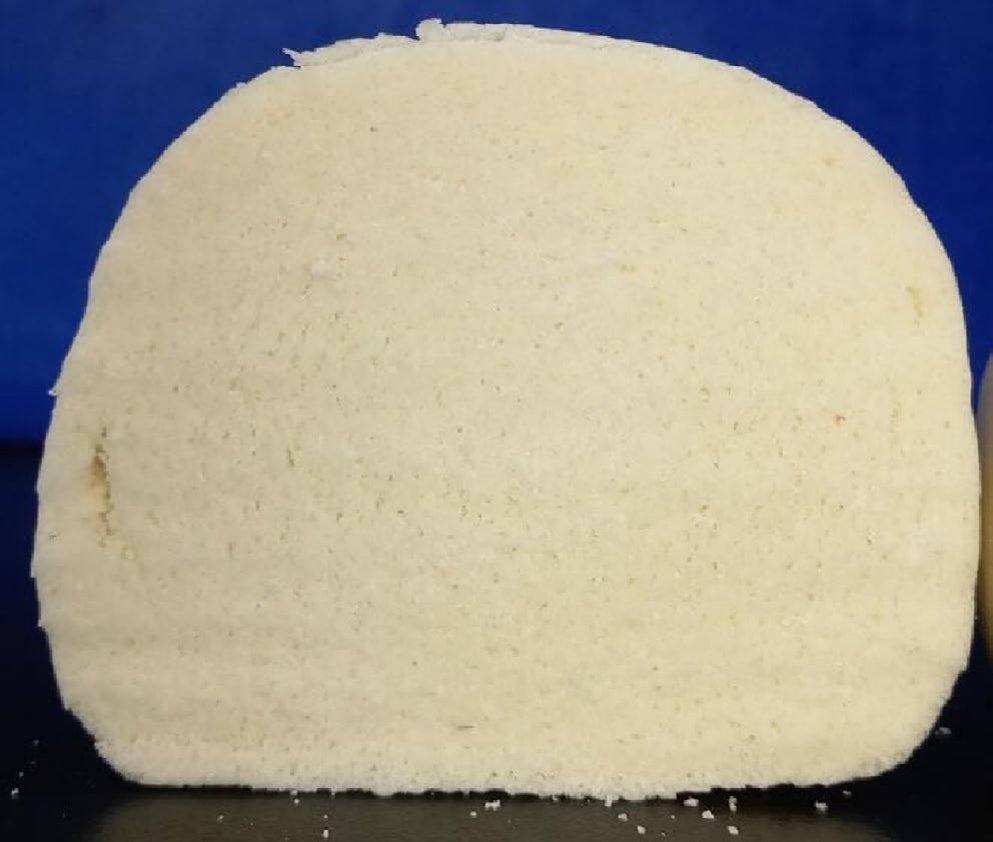
Photograph of the potato steamed bread

Afterward, the model validation was evaluated. As Table 7 shown, specific volume of the steamed bread at experiment A_2_B_3_C_3_D_3_ was 2.18 ml/g, and sensory score was 90.6. Specific volume of the steamed bread at experiment A_3_B_2_C_3_D_3_ was 2.21 ml/g, and sensory score was 92.7. Therefore, the optimal steamed bread preparation additions was 1.1% yeast, 1.4% NaHCO_3_, 0.75% citric acid, and 0.50% Ca(H_2_PO_4_)_2_. The prepared steamed bread had good sensory and texture properties. As Figure 2 shown, the prepared steamed bread possessed homogeneous interior texture, but looked a little yellow. The steamed bread also tasted a natural potato flavor.

**TABLE 7:** Verification test results

Combination	Hardness	Stickiness	Springness	Cohesiveness	Chewiness	Resilience	Specific volume (ml/g)	Sensory score
A_2_B_3_C_3_D_3_	4,654.31 ± 0.19	10.21 ± 0.023	0.91 ± 0.035	0.78 ± 0.051	3,303.41 ± 0.045	0.46 ± 0.006	2.18 ± 0.03	90.6 ± 0.6
A_3_B_2_C_3_D_3_	4,632.26 ± 0.22	9.47 ± 0.016	0.90 ± 0.031	0.79 ± 0.043	3,257.82 ± 0.052	0.45 ± 0.009	2.21 ± 0.02	92.7 ± 0.7

Note

Results are presented as mean value ± *SD*. Different letters within a column mean significant difference (*p* < .05).

## 4 CONCLUSIONS

As the rheological properties of the dough negatively related to the potato flour, the potato–wheat formulated flour with potato mass ratio of 35% was set as the basic flour. The addition of 6.5% wheat gluten could effectively improve rheological properties of the formulated flour. The L9(34) orthogonal design results showed that the optimal steamed bread preparation addition was 1.1% yeast, 1.4% NaHCO_3_, 0.75% citric acid, and 0.50% Ca(H_2_PO_4_)_2_. Therefore, the new potato steamed bread formula was 100% basic flour (potato/wheat mass ratio of 35:65), 6.5% wheat gluten, 1.1% yeast, 1.4% NaHCO_3_, 0.75% citric acid, and 0.50% Ca(H_2_PO_4_)_2_. The prepared steamed bread had good sensory and texture properties.

## CONFLICT OF INTEREST

All authors declare no conflicts of interest related to this article.

## ETHICAL APPROVAL

There was no ethical issue related to his manuscript.

## INFORMED CONSENT

Written informed consent was obtained from all study participants.

## Supporting information

Table S1
