# Supplementary material for: Preparation and quality evaluation of potato steamed bread with wheat gluten
Source: Food Sci Nutr. 2020 Jun 23;8(8):3989–98. doi: 10.1002/fsn3.1600 (PMC7455941; doi:10.1002/fsn3.1600)
Supplement: Supplementary file 1 — Table S1 [file FSN3-8-3989-s001.docx]

Table S1 Chemical composition characteristics of the formulated flour

| Sample | Water (%) | Ash (%) | Crude starch (%) | Crude protein (%) | Wet gluten (%) | Gluten index (%) |
| --- | --- | --- | --- | --- | --- | --- |
| S0 | 14.4±0.070^a^ | 0.42±0.004^f^ | 68.6±0.015^f^ | 11.6±0.014^a^ | 28.0±0.236^a^ | 59.8±0.724^d^ |
| S5 | 13.6±0.075^b^ | 0.47±0.056^e^ | 69.7±0.234^e^ | 11.4±0.210^b^ | 26.6±0.176^b^ | 69.8±2.067^bcd^ |
| S10 | 13.4±0.150^b^ | 0.49±0.006^e^ | 70.5±0.116^d^ | 11.3±0.106^b^ | 24.2±0.166^c^ | 64.4±3.045^bcd^ |
| S15 | 13.3±0.070^b^ | 0.51±0.007^d^ | 71.7±0.017^c^ | 11.0±0.007^c^ | 22.9±0.038^d^ | 61.9±1.583^cd^ |
| S20 | 12.8±0.350^c^ | 0.54±0.010^d^ | 72.0±0.013^c^ | 10.8±0.014^cd^ | 21.8±0.017^e^ | 64.8±2.936^bcd^ |
| S25 | 12.6±0.020^c^ | 0.57±0.004^c^ | 73.1±0.144^b^ | 10.7±0.134^de^ | 20.0±0.210^f^ | 70.6±0.132^bcd^ |
| S30 | 12.4±0.010^d^ | 0.58±0.012^c^ | 73.3±0.109^b^ | 10.6±0.099^e^ | 17.2±0.250^g^ | 73.6±0.992^b^ |
| S35 | 11.6±0.005^e^ | 0.60±0.003^b^ | 74.6±0.089^a^ | 10.5±0.099^f^ | 15.7±0.342^h^ | 72.2±2.553^bc^ |
| S40 | 11.4±0.002^f^ | 0.63±0.014^a^ | 75.2±0.014^a^ | 10.3±0.014^f^ | 12.9±0.102^i^ | 90.0±0.076^a^ |

Results are presented as mean values standard deviation of 3 replicates. Different letters within a column mean significant difference (p<0.05).
